# Predicting and Quantifying Antagonistic Effects of Natural Compounds Given with Chemotherapeutic Agents: Applications for High-Throughput Screening

**DOI:** 10.3390/cancers12123714

**Published:** 2020-12-10

**Authors:** G. Lavender Hackman, Meghan Collins, Xiyuan Lu, Alessia Lodi, John DiGiovanni, Stefano Tiziani

**Affiliations:** 1 Department of Nutritional Sciences, College of Natural Sciences, The University of Texas at Austin, Austin, TX 78712, USA; lavenderhackman@utexas.edu (G.L.H.); meghan.collins@austin.utexas.edu (M.C.); xlu@utexas.edu (X.L.); alessia@austin.utexas.edu (A.L.); 2 Department of Pediatrics, Dell Medical School, The University of Texas at Austin, Austin, TX 78723, USA; john.digiovanni@austin.utexas.edu; 3 Division of Pharmacology and Toxicology, College of Pharmacy, The University of Texas at Austin, Austin, TX 78712, USA; 4 Department of Oncology, Dell Medical School, LiveSTRONG Cancer Institutes, The University of Texas at Austin, Austin, TX 78723, USA

**Keywords:** natural products, cancer, chemotherapy, antagonism, synergy, drug screening

## Abstract

**Simple Summary:**

Increasing numbers of cancer patients are turning to complementary and alternative medicines (CAM) to facilitate or replace their cancer treatments, or they are obtaining natural products in their diet. This is concerning, as there is evidence of molecular interactions between these bioactive compounds and cancer drugs that can impede or reverse their efficacy and prevent cancer regression. High-throughput drug screening and deep learning techniques have successfully been applied in the past to evaluate synergistic cancer drug and natural product combinations. However, these techniques should be applied more commonly in the context of drug antagonism to uncover potentially harmful interactions and drive safer recommendations for cancer patients. In this review, we evaluate the antagonistic interactions between natural products and chemotherapeutics and highlight how the application of high-throughput screening and deep learning techniques can strengthen this area of research.

**Abstract:**

Natural products have been used for centuries to treat various human ailments. In recent decades, multi-drug combinations that utilize natural products to synergistically enhance the therapeutic effects of cancer drugs have been identified and have shown success in improving treatment outcomes. While drug synergy research is a burgeoning field, there are disagreements on the definitions and mathematical parameters that prevent the standardization and proper usage of the terms synergy, antagonism, and additivity. This contributes to the relatively small amount of data on the antagonistic effects of natural products on cancer drugs that can diminish their therapeutic efficacy and prevent cancer regression. The ability of natural products to potentially degrade or reverse the molecular activity of cancer therapeutics represents an important but highly under-emphasized area of research that is often overlooked in both pre-clinical and clinical studies. This review aims to evaluate the body of work surrounding the antagonistic interactions between natural products and cancer therapeutics and highlight applications for high-throughput screening (HTS) and deep learning techniques for the identification of natural products that antagonize cancer drug efficacy.

## 1. Introduction

Cancer is a general term used to describe a group of diseases that exhibit abnormal cell growth and proliferation. Second only to cardiovascular disease, cancer is the most common cause of death in the United States and remains a leading cause of death worldwide [1,2]. As precision medicine has become more prevalent, cancer treatments are increasingly cancer type- and individual-specific and often involve targeting multiple oncogenic molecular pathways with combinations of therapeutic agents [3,4]. However, the development of drug resistance and the toxicity and adverse secondary side effects of these treatments remains an obstacle for optimizing patient outcomes and quality of life [3,5]. As described in detail in the following section, synergistic combinations of drugs that work together to enhance therapeutic efficacy are often used to overcome these challenges. These combinations may include natural compounds, which are an attractive treatment option due to their ability to prevent cancer development and progression by inhibiting cancer metastasis, angiogenesis, proliferation, and differentiation, regulating autophagy, and inducing apoptosis with increased therapeutic efficacy and reduced general toxicity [6,7,8,9,10,11]. 

Bioactive natural compounds, including phytochemicals, such as polyphenols and flavonoids, vitamins, and other micronutrients, are obtained from plants, fungi, and bacteria, and other living organisms. Some are present in our food, herbs, and spices, while others are extracted from non-food sources that we would not normally obtain otherwise [12]. Bioactive natural products also form the basis for many types of complementary and alternative medicines (CAM), an umbrella term that is used to describe medical treatments and practices that are not a part of standard medical care but rather used alongside or potentially instead of it to facilitate healing, prevent disease recurrence, or alleviate adverse side effects [13]. Bioactive compounds are not limited to CAM applications and are also heavily used in standard medicine. Indeed, bioactive products have been used for over 60 years in the treatment of cancer, and approximately 60% of chemotherapeutic agents currently in use today are natural products or natural product derivatives [6]. Many of them, including curcumin from turmeric, resveratrol from red wine, isothiocyanates from cruciferous vegetables, and quercetin and ursolic acid from apples have been shown to have profound anti-tumor activity [14,15,16,17,18,19]. 

**Figure 1:**
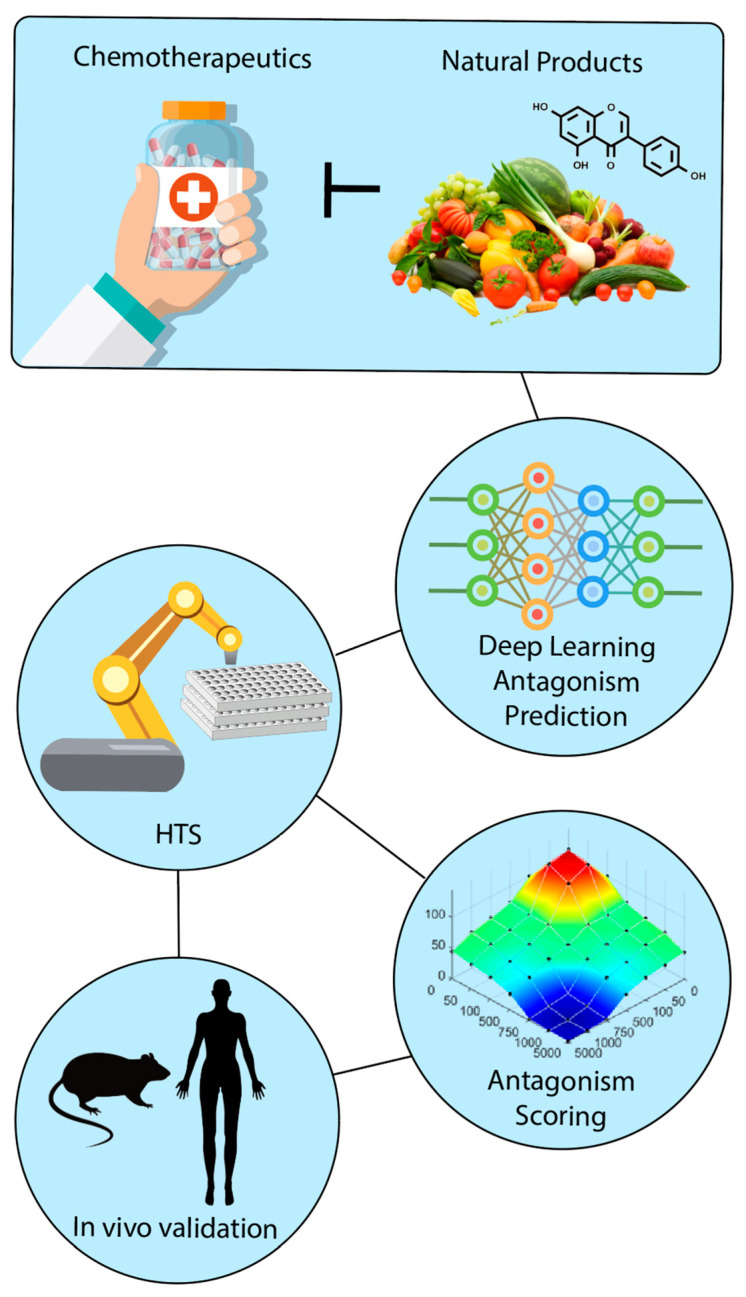
Natural product antagonism of chemotherapeutics can be predicted and quantified through the application of high-throughput screening techniques and deep learning that allow for mathematical quantification of antagonism and prioritization of combinations for further in vivo analysis and validation.

On the other hand, many common CAM treatments and natural products may be ineffective [20]. Worse, they can produce unintended adverse side effects or alter the actions of conventional medical treatments by inhibiting or amplifying their effects to dangerous levels [20,21,22]. For example, it is well known that furanocoumarins from grapefruit juice can interact with over 85 commonly used drugs, including statins, antiretroviral medications, and antihypertensive medications, and cause serious side effects by interacting with the cytochrome P450 3A4 enzyme and altering drug metabolism and bioavailability [23]. Because these adverse events are severe and can cause rhabdomyolysis, nephrotoxicity, heart issues, and even death, patients taking these medications are advised to avoid grapefruit altogether. While CAM and natural products can have serious, acute negative health effects, they may also be significantly less severe and present as subtle perturbations in health status or even go unnoticed [24]. As discussed in later sections, some natural products, including those found commonly in the Western diet such as fruits, vegetables, and green tea extracts, can directly interact with and antagonize conventional drugs and prevent them from exerting their desired treatment effects without causing overt adverse health events [20]. Table 1 includes a list of natural products, most of which are naturally present in foods that are common in the diet that can directly inhibit the molecular actions of chemotherapeutic agents in cancer models. Many of the natural products found in Table 1 are also considered to be components of CAM and touted as anti-cancer compounds, which may encourage individuals with cancer to supplement with these compounds in high amounts [20,25]. This is a growing concern, as up to 52% of cancer patients report using CAM before being diagnosed with cancer, and approximately 60% report using CAM after diagnosis and are often reluctant to notify their doctor of their CAM use [26,27,28]. Even cancer patients who do not partake in CAM use or supplement with natural products can still obtain potentially harmful bioactive natural products through the diet that may impede their treatment. Because CAM, natural products, and food and dietary supplements are all generally regarded as safe by cancer patients, they are more likely to consume natural products and supplements as complementary treatments and less likely to report adverse health events related to their usage [29]. This information, taken together with the fact that the sale and use of CAM and natural products are not subject to governmental regulation in many countries, represents a significant challenge to the safe and efficacious use of natural products concurrently with cancer treatments [22,26]. As outlined in Figure 1, this review aims to describe the body of work surrounding the inhibitory or antagonistic effects of natural products on chemotherapeutics in pre-clinical research and highlight high-throughput screening (HTS) and deep learning as essential tools that can be used to improve this field.

## 2. Drug Synergy and Antagonism

Synergy is generally defined as a combined treatment effect that is greater than the additive effect, or the sum of the individual effects of two non-interactive compounds, and indicates that the combined components “work together” to enhance the treatment [49]. Analogously, antagonism represents a combined treatment effect that is less than the additive effect, meaning that the combined components oppose each other [49]. For any two given drugs, the quality of synergism, antagonism, or additivity is highly dependent on the relative and absolute concentrations of the drugs and can change with increasing or decreasing concentrations [50]. Drug synergy research is based on the principle that drugs used in combination to treat a disease, as opposed to single-drug therapies, can improve the therapeutic effect of treatment by acting on the diseased cells via more than one molecular mechanism [17,51]. This can lead to lower toxicity and less adverse side effects by requiring lower doses of the drugs, and prevent the development of drug resistance. Ideally, a drug combination will both synergize to amplify the desired effect (i.e., lower proliferation, tumor shrinkage, enhanced apoptosis, etc.) due to increased efficacy while lowering the undesirable effects (i.e., toxicity to non-cancerous tissues, pain, etc.) due to increased potency [52]. Traditional synergy models do not take into account this difference between the potency and the efficacy of a drug combination and often conflate the two [53]. 

Drugs may interact via several mechanisms. These include pharmacokinetic interactions in which compounds can alter the absorption, metabolism, distribution, and clearance of drugs; and pharmacodynamic interactions, the focus here, whereby compounds achieve a physiological effect by acting on one or more molecular targets or pathways [54,55,56,57,58,59,60]. Unfortunately, clinical drug–drug or drug–nutrient interaction studies primarily focus on the pharmacokinetic components and adverse side effects of drug interactions and often overlook the pharmacodynamics and molecular actions [61]. As the use of natural products becomes more prevalent both in and out of clinical settings, it is increasingly important to fully characterize the potentially harmful and antagonistic effects of these compounds on cancer therapies, including their impacts on cellular growth, metabolism, and resistance mechanisms, to ensure their safe and efficacious use.

In recent years, combinations of natural products with cancer drugs that exhibit synergy have been developed to further improve upon existing treatment strategies due to the propensity of some natural products to provide improved therapeutic efficacy or overcome drug resistance with decreased risk for adverse side effects and toxicity in normal cells [9,10,17,62]. However, drug synergy research has been marked by conflicting principles and a failure to reach consensus on the appropriate way to define and quantify drug synergy, additivity, and antagonism [51,52,60,63,64,65]. Though this disagreement has persisted for nearly a century, drug synergy research remains an attractive and growing field that capitalizes on the ability of natural products to synergize with traditional cancer therapeutics [17]. The body of research on the topic of natural product and chemotherapeutic synergy is reviewed extensively elsewhere [17]. In contrast, relatively few studies have been done to assess the potential antagonistic effects of natural products on conventional chemotherapy drugs and how they may impede cancer therapy. Of the studies that report inhibitory interactions between natural products and chemotherapeutics in cancer models, the vast majority do not include a mathematical or statistical basis for the claim of “antagonism”, as emphasized in later sections. This area of research could be significantly improved by the standardization and widespread use of the appropriate mathematical foundations of synergy, additivity, and antagonism, and quantification of antagonism for each drug interaction bearing the claim “antagonistic”.

**Figure 2:**
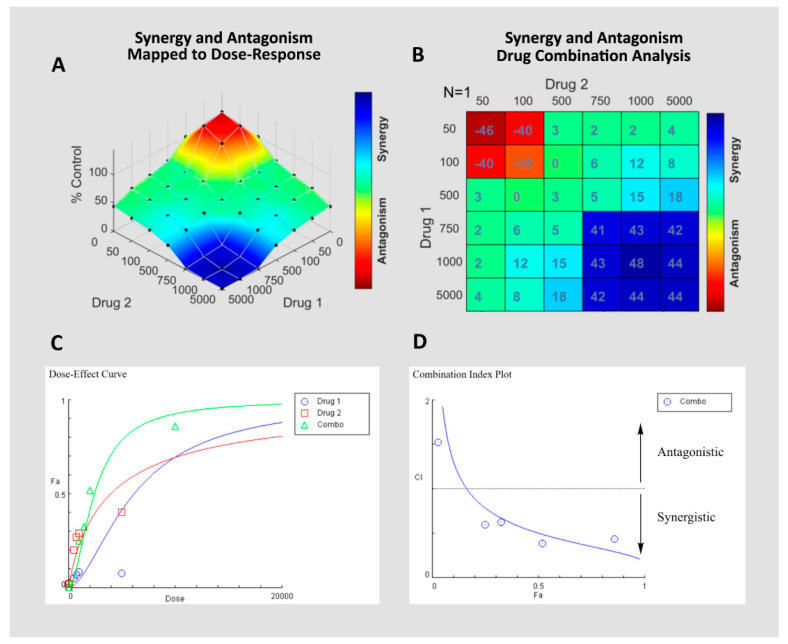
Examples based on mock data of a Loewe surface plot (**A**) and synergy matrix (**B**) generated with Combenefit, and dose–effect curve (**C**) and Chou–Talalay combination index plot (**D**) generated with CompuSyn.

Multiple mathematical models have been developed to quantify drug combination synergy, antagonism, and additivity. Herein, we report the most commonly utilized models and their limitations (Table 2). These and many other models for drug synergy have been reviewed extensively elsewhere [49,52,53,63]. While drug combination data can be analyzed using personalized scripts in Matlab, R, and other computing environments based on the equations outlined in Table 2, several software packages have been developed to visualize drug combination data and quantify synergy, additivity, and antagonism based on univariate or bivariate data: CompuSyn (version 1.0, https://www.combosyn.com/index.html), Combenefit (version 2.021, https://sourceforge.net/projects/combenefit/), SynergyFinder (version 2.0, https://synergyfinder.fimm.fi/), and CImbinator (version 1.0, https://bio.tools/cimbinator). [51,66,67,68]. Example outputs from these software programs are included in Figure 2. In the following section, we describe natural products that inhibit the activity or outcomes of chemotherapeutics and indicate whether a mathematical model has been used to support and quantify antagonism in each case.

## 3. Natural Products that Inhibit Chemotherapeutics

### 3.1. Genistein

Genistein is an isoflavonoid derived from soybeans and soy-containing products, such as tofu, as well as fava beans and kudzu. Genistein supplementation (a form of CAM) is commonly used as a hormone replacement therapy alternative for postmenopausal women [70]. It is classified as a natural xenoestrogen, or a chemical that mimics estrogen in the body. Genistein has been shown to exhibit potent anticancer effects and synergize with certain cancer therapeutics [71,72,73]. However, it has also been shown to directly inhibit the anti-cancer activity of the chemotherapy drugs tamoxifen, letrozole, and palbociclib/letrozole combination therapy in breast cancer models [30,31,32,33].

Genistein was reported to prevent the anti-cancer activity of the aromatase inhibitor letrozole and stimulated tumor growth both alone and in combination with letrozole therapy in an MCF-7Ca xenograft model at concentrations that were consistent with dietary exposure levels [31]. Concentrations of genistein relevant to the human diet were also shown to inhibit the anti-cancer activity of the antiestrogen drug tamoxifen in ovariectomized nude mice bearing MCF-7 breast cancer tumors [31]. Additional in vivo and in vitro data support that low doses of genistein negate the growth inhibitory effects of tamoxifen in the tumors of MMTV-neu/ErbB2 transgenic mice and MCF-7 cells [33]. However, Mai et al. (2007) reported a synergistic induction of apoptosis following the administration of the combination of genistein and tamoxifen in a different breast cancer cell line (BT-474) [74]. Warth et al. (2018) found that concentrations representative of food consumption of genistein and the estrogenic mycotoxin zearalenone both reversed the anti-oncogenic effects of combination letrozole and palbociclib therapy in MCF-7 breast cancer cells by counteracting the metabolic aberrations induced by the combination treatment [32]. These findings are especially relevant as the palbociclib/letrozole drug combination was granted accelerated US FDA approval for the treatment of estrogen receptor-positive breast cancer in 2015 [32]. These findings did not include the quantification of antagonism using a mathematical or statistical model. 

### 3.2. (-)–Epigallocatechin Gallate (EGCG)

(-)–Epigallocatechin gallate (EGCG) is the predominant bioactive polyphenol present in green tea. It is also present in smaller amounts in other types of tea, berries, pears, apples, avocadoes, and some stone fruits. Green tea extract is widely available for purchase as a highly concentrated supplement and is a very commonly used form of CAM due to its low toxicity and health benefits, including neuroprotective, cardioprotective, and anti-cancer effects [75,76,77,78,79,80,81]. However, EGCG was found to directly inhibit the tumor-suppressive activity of the proteasome inhibitor bortezomib in in vitro and in vivo models of multiple myeloma, and in vitro models of prostate cancer and glioblastoma [34,35,36].

Golden et al. (2009) found that EGCG, as well as various other green tea polyphenols, and complete green tea extract blocked the cytotoxic effects of bortezomib in RPMI/8226 and U266 multiple myeloma cell lines and LN229 glioblastoma cells by direct chemical interaction with the drug at concentrations that were realistically achievable through the diet [35]. EGCG was also shown to negate the anti-tumor effects of bortezomib in nude mice implanted with RPMI/8226 multiple myeloma tumors [35]. Kim et al. (2009) quantified the antagonistic effect of EGCG on bortezomib treatment in U266, RPMI/8226, and MC/CAR multiple myeloma cell lines using the Chou Talalay method combination index (CI). They found that EGCG directly antagonized the cancer growth inhibition of bortezomib in U266, RPMI/8226, and MC/CAR with CI values at ED-90 of 3.02, 3.99, and 4.01, respectively [36]. EGCG was also shown to inhibit bortezomib activity at concentrations representative of human serum in PC3 prostate cancer cells via direct binding and the inhibition of bortezomib [34]. However, a pre-clinical study found that EGCG only inhibited bortezomib activity in vivo at concentrations 80-fold higher than those achieved through supplementation [82].

### 3.3. Curcumin

Curcumin is the predominant diarylheptanoid in turmeric, a spice derived from the perennial plant *Curcuma longa*. Curcumin is what provides the characteristic yellow pigment in turmeric, which is commonly used in Indian and Southeast Asian cuisines and medicines, and celebrated for its efficacy in treating wounds, inflammation, digestive issues, and some cancer types [83]. In recent years, supplementation with curcumin has gained popularity in Western nations due to emerging evidence that it is a potent chemopreventive and chemotherapeutic agent [84]. Indeed, curcumin has shown efficacy in inhibiting the growth and development of many cancer types, including prostate, breast, colorectal, hepatic, and lung cancers, among several others [15,84,85,86,87,88]. Even so, there have been reports that curcumin has the potential to inhibit the activity of certain anticancer agents in vitro [37,38,39,89]. 

Curcumin, at realistic concentrations representative of human serum after ingestion, was found to inhibit MCF-7, MDA-MB-231, and BT-474 breast cancer cell apoptosis mediated by chemotherapy drugs, camptothecin, mechlorethamine, and doxorubicin, by preventing the generation of reactive oxygen species (ROS) [39]. Curcumin also blunted the tumor growth inhibition of cyclophosphamide in mice bearing BT-474 breast cancer tumor xenografts [39]. However, Ma et al. (2017) reported that micellar co-delivery of curcumin combined with doxorubicin synergistically improved anti-tumor efficacy in MCF-7/Adr cells and 4T1 tumors [90]. Saleh et al. (2012) quantified the antagonistic interaction between curcumin and the topoisomerase inhibitor etoposide using isobologram analysis and found that the co-treatment of curcumin with etoposide significantly reduced the cytotoxicity of both agents in MCF-7 breast cancer cells, HepG2 liver cells, HCT116 colorectal cancer cells, and HeLa cervical cancer cells with interaction index values of 2.5, 3.3, 67, and 19, respectively [37]. In contrast, the curcumin and etoposide combination treatment exhibited a synergistic interaction in U251 glioblastoma cells with an interaction index value of 0.59 [37]. Curcumin was also shown to inhibit apoptosis mediated by both camptothecin and etoposide in Hep3B liver cancer cells [38].

### 3.4. Vitamin C

Vitamin C is an essential vitamin present in citrus fruits, potatoes, red and green peppers, broccoli, tomatoes, and other foods, and is one of the most widely consumed supplements in the United States [91]. Vitamin C is used for a variety of health benefits, most commonly related to immunity and wound healing, and there is emerging evidence that vitamin C may be able to aid in cancer treatment [92]. However, vitamin C has been found to directly inhibit many chemotherapeutics across several cancer types both in vivo and in vitro [40,41,42,43]. 

Vitamin C has been shown to directly bind and inactivate bortezomib and prevent its anti-cancer activity in prostate, breast, oral, cervical, endometrial, and lung cancer cell lines at concentrations representative of human serum [41,43]. Perrone et al. (2009) found that vitamin C inhibited the activity of bortezomib on MM1S multiple myeloma cells as well as RPMI/8226 multiple myeloma xenografted severe combined immune deficient (SCID) mice [42]. They also showed that plasma taken from healthy volunteers given 1 g per day of vitamin C was able to negate the cancer growth suppression of bortezomib in RPMI/8226 cells in vitro [42]. Clinically relevant concentrations of vitamin C have also been demonstrated to reduce the cytotoxicity of multiple chemotherapeutic agents, including vincristine, doxorubicin, methotrexate, cisplatin, and imatinib mesylate in K562 chronic myeloid leukemia cells and RL lymphoma cells by preserving the membrane potential of the mitochondria [40]. Vitamin C also negated the anti-cancer effects of doxorubicin in vivo in mice with RL lymphoma xenografted tumors [40]. These findings did not include the quantification of antagonism using a mathematical or statistical model.

### 3.5. Tangeretin

Tangeretin is a dietary flavonoid found in the peels of citrus fruits, such as tangerines, mandarins, oranges, and grapefruits. It is also a major constituent of citrus bioflavonoid supplements (a form of CAM) that are frequently used for their antioxidant and anti-inflammatory activity [93]. Tangeretin was reported to negate the anti-cancer effects of tamoxifen in MCF-7/6 breast tumor bearing mice when added to the drinking water along with tamoxifen and reduced their median survival time compared to those that received tamoxifen alone [44]. However, this effect was not replicated in vitro, as tangeretin was shown to enhance cancer growth inhibition in MCF-7/6 cells [44]. This discrepancy is likely due to the ability of tangeretin to downregulate natural killer cells and prevent the elimination of tumor cells in vivo [45]. These findings did not include the quantification of antagonism using a mathematical or statistical model.

### 3.6. Xanthorrhizol

Xanthorrhizol is a sesquiterpenoid derived from the rhizome of *Curcuma xanthorriza*, also called Javanese turmeric in Indonesia, Thailand, and Malaysia, where it has been used for its medicinal properties for many years. It is available for purchase as a supplement both alone and in combination with curcumin and other bioactive compounds. Xanthorrhizol has been shown to have many anti-cancer effects, including its ability to inhibit the proliferation of colon cancer cells in vitro and prevent metastasis and tumor promotion in mouse models of skin and lung cancer [94,95,96,97]. However, there is evidence that repeated dosing of xanthorrhizol can negate the tumor-suppressive ability of tamoxifen in MCF-7 implanted nude mice, though this interaction was not seen in the MCF-7 in vitro model [46]. These findings did not include the quantification of antagonism using a mathematical or statistical model.

### 3.7. Si-Wu-Tang (SWT)

Si-Wu-Tang (SWT) is a form of traditional Chinese medicine comprised of the combined extracts from four herbs: *Radix Paeoniae Alba* (bai shao yao), *Rhizoma Ligusticum Chuanxiong* (chuan xiong), *Radix Angelica Sinensis* (dang gui), and *Radix Rehmanniae Preparata* (shu di huang). It has been used to treat gynecological disorders and support menstrual health since the 12th century [98]. SWT supplementation has also gained popularity in Western nations to support women’s health and is available for purchase in the United States from multiple online retailers. Despite its claimed health benefits, SWT has been shown to stimulate the proliferation of breast cancer and reverse the anticancer effects of the antiestrogen chemotherapy drug, tamoxifen, in both in vitro and in vivo MCF-7 breast cancer models [48,99]. SWT also reversed the antiproliferative effects of the monoclonal antibody drug, trastuzumab, in SK-BR-3 and BT-474 breast cancer cells [48]. These findings did not include the quantification of antagonism using a mathematical or statistical model.

### 3.8. Quercetin and Myricetin

Quercetin and myricetin are dietary flavonoids that are present in a diverse array of fruits and vegetables, including apples, onions, red grapes, berries, and herbs. Both are widely available for purchase, either alone or in combination with other bioactive compounds. Quercetin and myricetin are well known for their potent antioxidant activity, and both have been shown to exhibit anti-cancer activity via proteasome inhibition and the induction of apoptosis in several cancer types in vitro [100,101,102,103]. Even so, there is evidence that quercetin and myricetin prevent the anti-cancer and pro-apoptotic activity of the proteasome inhibitor, bortezomib, in chronic lymphocytic leukemia primary cells and several malignant B cell lines [47]. Quercetin was also shown to strongly antagonize the activity of bortezomib (based on the Chou Talalay combination index) in MC/CAR, U266, and RPMI/8226 multiple myeloma cell lines with CI values at ED–90 of 5.04, 3.70, and 5.27, respectively, as well as primary myeloma cells by direct chemical interaction with the drug [36]. 

### 3.9. Tannic Acid, Gallic Acid, Caffeic Acid

Tannic acid, gallic acid, and caffeic acid are natural phenolic compounds present in a wide variety of grains, fruits, vegetables, and beverages, including coffee, wine, tea, berries, and herbs, along with many additional sources. They are all also widely available for purchase in the form of supplements, both individually and in various combinations. Kim et al. (2009) found that these compounds were able to directly interact with bortezomib and antagonize its anti-cancer activity (based on the Chou Talalay combination index) in MC/CAR, U266, and RPMI/8226 multiple myeloma cell lines, as well as primary myeloma cells [36]. The CI values at ED–90 for tannic acid, gallic acid, and caffeic acid, respectively, were 27.24, 6.94, and 4.97 for MC/CAR, 6.70, 3.08, and 4.22 for U266, and 11.39, 3.34, and 8.54 for RPMI/8226 [36].

## 4. Tools for Evaluating or Predicting Drug Synergy and Antagonism and Their Applications

Techniques commonly used in pre-clinical settings to study drug synergy, such as high-throughput screening, can also be used to study antagonistic interactions between natural products and CAM and cancer drugs, often within the same methodology and utilizing the same mathematical parameters [104]. Additionally, recent innovations in deep learning models have made it possible to predict drug interactions with great accuracy and have the potential to be applicable to natural product and chemotherapeutic drug antagonism research in the future. 

### 4.1. High Throughput Screening for Natural Product–Drug Antagonism

High-throughput screening (HTS) is a biomedical research technique that allows for the rapid analysis of the biological activity of chemical substances, alone or in combination, on a particular cellular model or pathway in 2-dimensional cell cultures [105]. As automation has improved in accuracy and precision, microplates have been developed with smaller and more dense wells (96-, 384-, 1536-, and even 3456-well plates are in use today) so that tens to hundreds of thousands of compounds can be tested in a single day. In HTS experiments, drugs may be combined with other drugs or bioactive compounds, and the effect on the cells can be quantified relative to controls via mathematical models for drug synergy, as previously described. 

A limitation of in vitro cancer models for drug combination screening is that they often generate results that are not reproducible in in vivo models. However, in vitro methods for evaluating drug interactions, while they are limited in their ability to accurately represent the disease environment and conditions, are preferred over in vivo disease models for HTS techniques, due to their feasibility for testing multiple drug concentrations, low cost, and rapid completion time [106]. It has been shown that the utilization of a culture medium that more accurately mimics the tumor microenvironment for in vitro assays can improve their concordance with in vivo models of disease and reduce unwanted environmental features that result from the culturing of tissues ex vivo [107]. The use of primary, patient-derived tissues or implementation of co-culturing methods for screening experiments can also help to reduce disparities between in vitro and in vivo results [108]. It is important that in vitro drug combinations that exhibit synergy or antagonism be used not as absolute proof of drug interaction effects but to identify drug combinations that warrant further mechanistic investigation, which may include metabolomics, proteomics, genomics, and in vivo experiments for validation [60,109].

In pre-clinical cancer drug development, the utilization of HTS to assess the ability of natural products to amplify the anti-cancer effects of chemotherapeutics, or produce synergy, is becoming increasingly common. However, likely due to the relatively lower interest in natural product antagonism of chemotherapeutic drugs, these techniques are often underutilized to study natural product-mediated drug antagonism in cancer, although it can be undertaken in the same experiments and utilize the same mathematical models. HTS is especially conducive to the study of drug interactions involving CAM, as many alternative medicines are comprised of multiple active compounds used in combination, which can be applied separately or in concert in HTS experiments to elucidate the individual effects as well as the interactions between the compounds in the CAM itself. This is also unique in that it can employ the use of libraries of bioactive natural compounds and identify previously unknown inhibitors of chemotherapeutics across multiple types of cancer cell lines as well as assess their impact on normal cell lines [105]. The lack of application of HTS to study natural product–drug antagonism is likely worsened by the fact that no single mathematical framework has been established to allow for standardized quantification of antagonism in in vitro models. Even so, HTS techniques and implementation of mathematical models to support claims of antagonism between natural products and chemotherapeutics should be more commonly utilized in the study of drug–nutrient interactions. This unbiased methodology for identifying unwanted and potentially deleterious herb–drug, food–drug, or nutrient–drug interactions could significantly improve the safe and efficacious use of chemotherapeutics and potentially lead to improved therapeutic outcomes in cancer patients. 

### 4.2. Deep Learning for Prediction of Natural Product Antagonism

As natural product libraries grow in size and it becomes increasingly infeasible to investigate every possible combination of natural products with chemotherapeutics due to cost and time restraints, it can be beneficial to employ a framework for predicting synergistic or antagonistic interactions. As described in detail by Adam et al. (2020), several methods exist for predicting combination therapy interactions, and deep learning techniques are at the cutting edge of this research [110]. Deep learning is a subcategory of machine learning that employs algorithms to create multiple layers of artificial neural networks (ANNs) that mimic the structure of the human brain and synthesize large data sets to create highly powerful predictive tools [111]. The application of these emerging tools in natural product drug combination studies could accelerate the identification of natural products that antagonize chemotherapeutics and allow for a much more robust understanding of potentially harmful natural product–drug interactions.

Xia et al. (2018) developed a deep learning technique to predict synergistic combination drug interactions based on molecular features, including gene expression, microRNA expression, protein abundance, and drug descriptors and fingerprints [112]. This is based on the NCI-ALMANAC, a drug screening resource that includes growth inhibition data on 5000 pairs of FDA approved cancer drugs in a panel of 60 cancer cell lines (NCI-60) [113]. This model is highly accurate, and explained 94% of the response variance, with much of the predictive power coming from drug descriptor data [112]. However, it is not yet applicable to new drugs or natural products and other cell lines and tumor samples. 

DeepSynergy is another deep learning tool that implements a feed-forward neural network to model drug combination effects based on cancer cell genomic profiles and chemical profiles of drugs [114]. DeepSynergy performed well compared to other machine learning tools to predict synergistic combinations (AUC of 0.90) and was 7.2% more accurate than their second-best model [114]. While this approach is highly accurate, it is limited in its inability to provide predictions based on novel drugs and cancer cell lines and would require additional data and updated algorithms to be applied to the natural product and chemotherapy synergy and antagonism research.

Currently, there are several more tools available to quantify and visualize synergy than tools to predict synergy [110]. Moreover, the majority of these models are intended for the quantification or prediction of synergistic or greater-than-additive responses rather than antagonistic or lower-than-additive responses. The development of these tools is at the forefront of drug discovery innovation and they are still relatively new, and as such they will require additional datasets, input variables, and algorithm optimization in the future to further bolster their predictive capacity and applicability for new drugs and cell types. It is critical that future developments in deep learning models for drug combination prediction prioritize antagonistic interactions as well as synergistic interactions and permit the analysis of natural products and other novel bioactive compounds. This will allow for accelerated identification of antagonistic drug interactions with natural products and has the potential to help improve treatment outcomes and prevent poor treatment response in cancer patients. 

## 5. Conclusions

Drug synergy research is a burgeoning field, despite disagreements regarding the mathematical definitions of synergy, additivity, and antagonism and issues with translatability between in vitro and in vivo systems. As natural products used in combination with traditional anti-cancer drugs become more common, and cancer patients continue to seek out alternative medicines outside of clinical recommendations, it is increasingly important to pinpoint and understand the potentially inhibitory and antagonistic impacts of natural products on the activity of chemotherapeutics. High throughput screening (HTS) is a relatively low-cost and expeditious technique used frequently to reveal synergistic drug interactions. However, HTS should be utilized more commonly to study antagonism between natural products and chemotherapeutics and bolster drug–nutrient interaction research in cancer. Advances in deep learning models that take into account natural products as well as conventional drugs will be essential for continued progress in this field. The identification of natural products that antagonize chemotherapeutics and the elucidation of their mechanisms of action have the potential to improve the safety and efficacy of chemotherapeutics, as they are commonly used in combination with various natural products and CAM, and can potentially help explain causes for drug failure or resistance. Improvements in this field can also help fuel better recommendations for the timing and use of natural products and CAM alongside chemotherapy and prevent potentially dangerous interactions.

## Figures and Tables

**Table 1:** Natural products that antagonize or inhibit chemotherapeutics.

Natural Product	Common Sources		Chemotherapy Drug	Cancer Type	Antagonism Mechanism	Antagonism Values (Cell Line–Value)	Ref
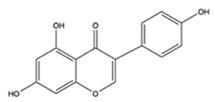 Genistein	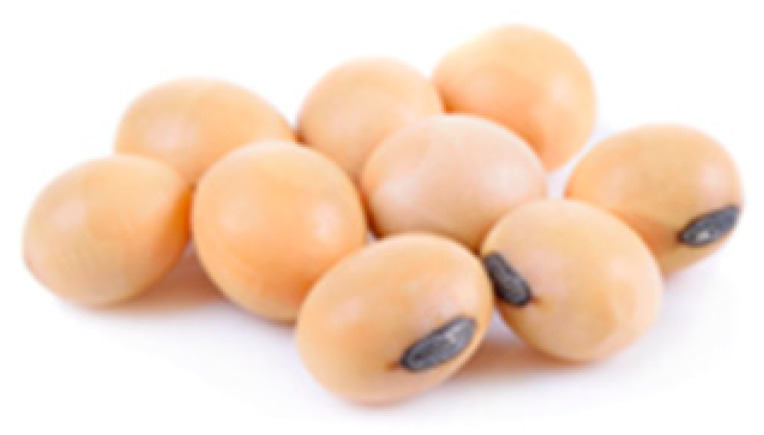	Soybeans, fava beans, kudzu	Tamoxifen, letrozole, palbociclib + letrozole	Breast cancer	Reversed the anti-cancer effects of tamoxifen by inducing increased expression of estrogen responsive and cell cycle proteins pS2, PR, and cyclin D1 [30,31]; and activating mTOR by preventing amino acid depletion induced by palbociclib + letrozole [32]	Undetermined	[30,31,32,33]
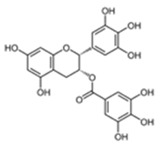 EGCG	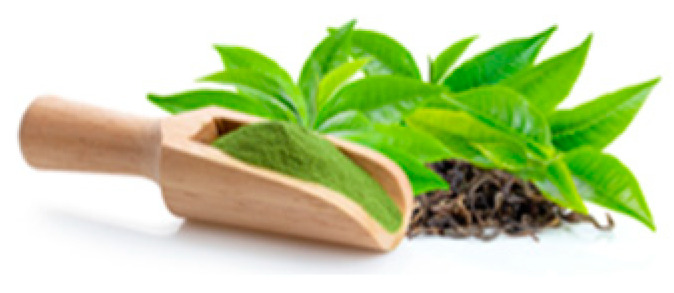	Green tea, berries, pears, apples, avocadoes	Bortezomib	Multiple myeloma, glioblastoma, prostate cancer	Protected against cancer cell death induced by bortezomib by preventing proteosome inhibition and ER stress induction and exacerbating autophagy activation to prevent apoptosis [34]; and by direct interaction with the drug’s boronic acid moiety that prevented proteosome inhibition [35,36]	U266-3.02 RPMI/8226-3.99 MC/CAR-4.01	[34,35,36]
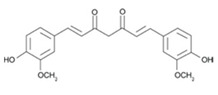 Curcumin	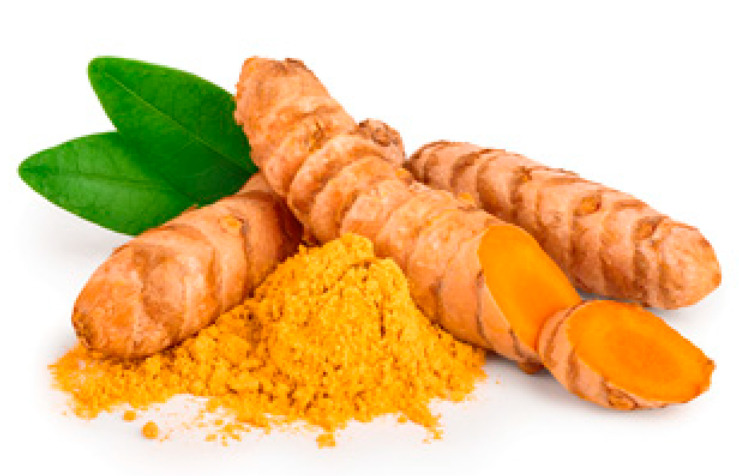	Turmeric	Etoposide, doxorubicin, mechlorethamine, camptothecin	Breast cancer	Prevented cancer cell death induced by etoposide and camptothecin by causing cell cycle arrest in the G1, S, or G2/M phases and allowing time for DNA repair prior to cell division [37,38]; and by inhibiting ROS generation and JNK activation induced by mechlorethamine and camptothecin [39]	MCF-7-2.5 HepG2-3.3 HCT116-67 HeLa-19	[37,38,39]
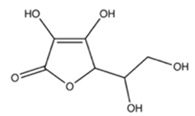 Vitamin C	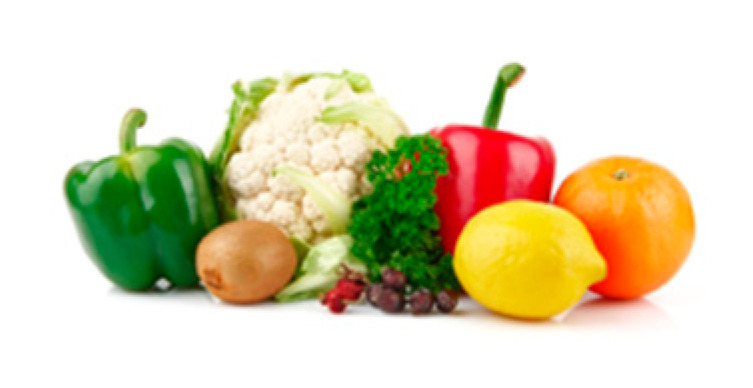	Citrus fruits, potatoes, red and green peppers, broccoli, cauliflower, tomatoes	Bortezomib, doxorubicin, vinicristine, methotrexate, cisplatin, imatinib mesylate	Multiple myeloma, chronic myelogenous leukemia, B-cell lymphoma, breast, prostate, lung, oral, endometrial, and cervical cancer	Inhibited the anti-cancer effects of vinicristine, doxorubicin, methotrexate, imatinib mesylate, and cisplatin by preserving mitochondrial membrane potential and preventing apoptosis [40]; and by forming a chemical complex with bortezomib and blocking its activity [41,42]	Undetermined	[40,41,42,43]
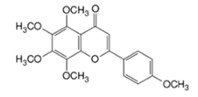 Tangeretin	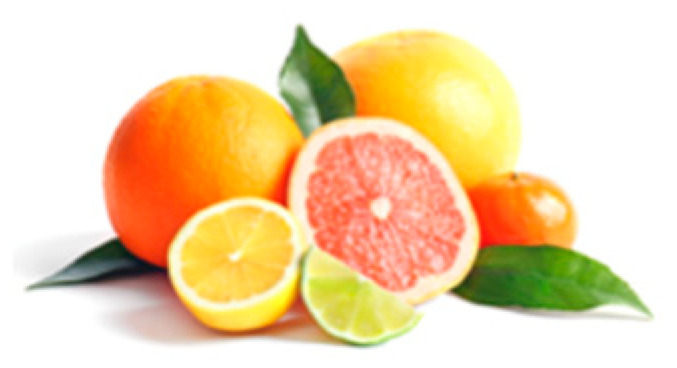	Tangerines, mandarins, oranges, grapefruits	Tamoxifen	Breast cancer	Inhibited the anticancer effects of tamoxifen by downregulating NK cells and preventing tumor cell elimination in vivo	Undetermined	[44,45]
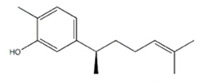 Xanthorrhizol	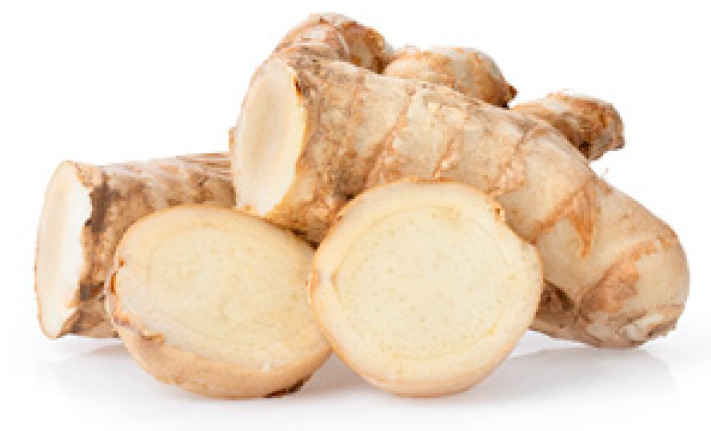	*Curcuma xanthorriza* (Javanese turmeric)	Tamoxifen	Breast cancer	May have reversed the anticancer effects of tamoxifen by activating the P38/MAPK pathway	Undetermined	[46]
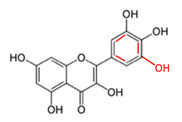 Quercetin and Myricetin	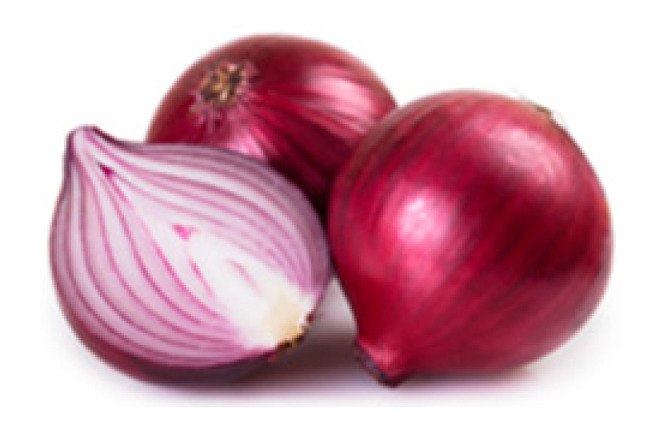	Onions, apples, grapes, berries, herbs	Bortezomib	B-cell lymphoma, chronic lymphocytic leukemia, multiple myeloma	Prevented the anticancer activity of bortezomib by directly interacting with the drug’s boronic moiety and inhibiting its activity [36,47]	U266-3.70 RPMI/8226-5.27 MC/CAR-5.04 (Quercetin)	[36,47]
Tannic acid, gallic acid, and caffeic acid	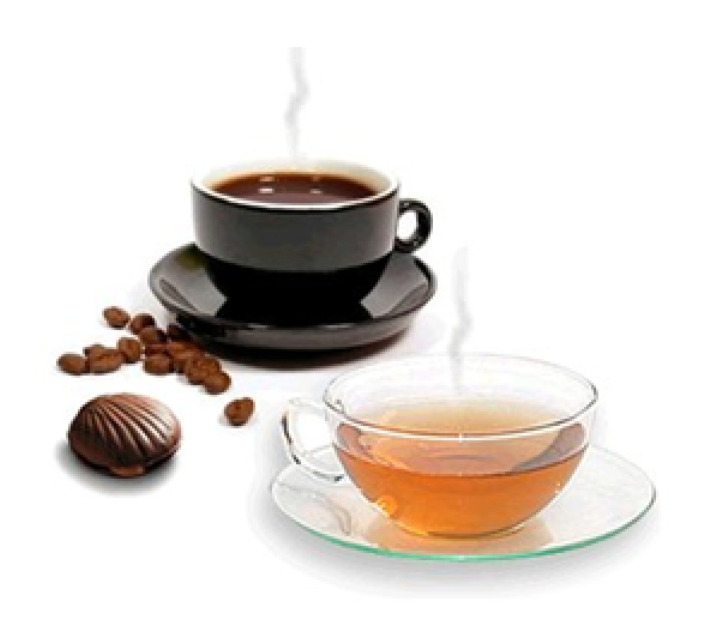	Coffee, tea, wine, grains, fruits, vegetables, berries, herbs	Bortezomib	Multiple myeloma	Blocked the anticancer activity of bortezomib by direct chemical interaction with its boronic acid moiety	U266- 6.70/3.08/4.22 RPMI/8226-11.39/3.34/8.54 MC/CAR-27.24/6.94/4.97 (TA/GA/CA)	[36]
Si-Wu-Tang	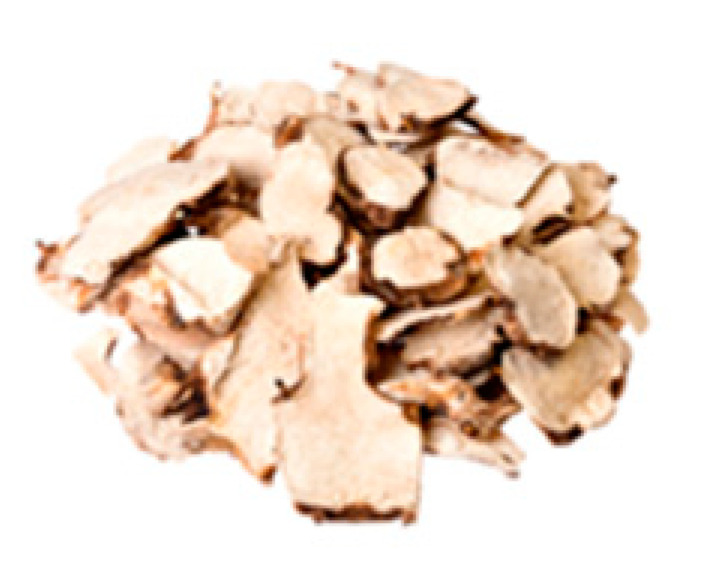	Combination: *Radix Paeoniae Alba* (bai shao yao), *Rhizoma Ligusticum Chuanxiong* (chuan xiong), *Radix Angelica Sinensis* (dang gui), and *Radix Rehmanniae Preparata* (shu di huang)	Tamoxifen, trastuzumab	Breast cancer	Reversed the cytotoxicity induced by tamoxifen by inactivating P27, and reversed the cytotoxicity of trastuzumab by activating AKT signaling and suppressing p27 by activating the P38/MAPK pathway	Undetermined	[48]

**Table 2:** Common mathematical models for drug synergy and their limitations [49,69].

Synergy/Antagonism Model	Features	Strategy	Limitations	Equation	Software(s)
Loewe Additivity	Constant potency ratio Equal individual maximum effects Sham compliant: a drug cannot exhibit synergy with itself	Dose-effect based	Requires dose-effect curves for each compound Constant potency ratio and equal maximum effects is unlikely	$\mathrm{Effect}\left(a+b\right)=E_{A}\left(a+a_{b}\right)=E_{B}\left(\;b_{a}+b\right)=E_{AB}\;$ $a+a_{b}=A\;↔a+b\times R=A\;↔a+b\times \frac{A}{B}=A\;\;$ $\frac{a}{A}+\frac{b}{B}=1$ $CI=\frac{a}{A}+\frac{b}{B}$	Combenefit SynergyFinder CImbinator
Bliss Independence	Drugs work by separate, non-overlapping mechanisms Assumes exponential dose-effect curves Expressed as a probability (0 ≤ $E_{AB}$ ≤ 1)	Effect-based	Most compounds do not have single, isolated mechanisms of action Exponential dose-effect curves unlikely	$E_{A}+E_{B}\left(1-E_{A}\right)=E_{A}+E_{B}-E_{A}E_{B}$ $0\;\leq E_{A}\leq 1\;and\;0\;\leq E_{B}\leq 1\;$ $CI=\frac{E_{A}+E_{B}-E_{A}E_{B}\;}{E_{AB}}$	Combenefit SynergyFinder
Highest Single Agent	A combined effect greater than that of the most effective single agent is synergistic Quantified by p-value of combined effect vs. highest individual agent	Effect-based	Fails to reflect true synergistic interactions (does not account for additivity)	$CI=\frac{\max\left(E_{A},E_{B}\right)}{E_{AB}}$	Combenefit SynergyFinder
Chou-Talalay	Based on the median effect equation derived from the mass action law Takes into account the Hill equation, Michaelis-Menten equation, Henderson-Hasselbalch equation, and Scatchard equations	Dose-effect based	Requires a dose-effect curve for each individual compound	$f_{a/}f_{u}={\left(D/D_{m}\right)}^{m}\;\left(median-effect\;equation\right)$ $CI=\frac{D_{1}}{E_{1}}+\frac{D_{2}}{E_{2}}\;$	CompuSyn

CI = Combination index. CI = 1 represents additivity, CI > 1 represents antagonism, and CI < 1 represents synergy. Drugs A and B administered at doses a and b give individual effects of E_A_ and E_B_ and a combined effect of E_AB_. R = potency ratio. a + a_b_ = dose of A giving effect E_AB_. b_a_ + b = dose of B giving effect E_AB_. f_a_ = fraction of cells killed. f_u_ = fraction of cells alive. m represents the dose-effect curve sigmoidicity. D_m_ = median-effect dose. D_1_ and D_2_ = actual experimental doses. E_1_ and E_2_ = expected doses to produce the observed effect.
